# Probiotics as a Coadjuvant Factor in Active or Quiescent Inflammatory Bowel Disease of Adults—A Meta-Analytical Study

**DOI:** 10.3390/nu12092628

**Published:** 2020-08-28

**Authors:** Manuel Pabón-Carrasco, Lucia Ramirez-Baena, Samuel Vilar-Palomo, Aurora Castro-Méndez, Raúl Martos-García, Isabel Rodríguez-Gallego

**Affiliations:** 1 Spanish Red Cross Nursing School, Universidad de Sevilla, Avda de la Cruz Roja nº 1 Dpdo, 41009 Seville, Spain; mpabon@cruzroja.es (M.P.-C.); rmartos@cruzroja.es (R.M.-G.); isroga@cruzroja.es (I.R.-G.); 2 Hospital Virgen del Rocío, Unidad de Anestesiología y Reanimación, Servicio Andaluz de Salud, Av. Manuel Siurot, SN., 41013 Seville, Spain; samuelvilarpalomo@hotmail.com; 3 Faculty of Nursing, Physiotherapy and Podology, Universidad de Sevilla, C/Avenzoar 6, 41009 Seville, Spain; auroracastro@us.es

**Keywords:** ulcerative colitis, Crohn’s disease, inflammatory bowel disease, efficacy, probiotics

## Abstract

(1) Background: Inflammatory bowel diseases are pathologies of unknown etiology and auto-immune pathogenia. The use of probiotics is studied in order to increase the arsenal of treatments. The aim was to assess the efficacy of the probiotics in these diseases in the active or quiescent phases; (2) Methods: A systematic review with meta-analysis was performed by an exhaustive bibliographic search in Medline, Cinahl, Embase, Scopus, Web of Science, and Cochrane Library. The inclusion criteria were studies of more than 10 years, English/Spanish, clinical trials, and involving human beings. Relative risk was used to compare efficacy, which was meta-analyzed using a fixed effects model. Heterogeneity was evaluated with the Higgins I^2^ test; (3) Results: Nineteen studies were included in the systematic review and 17 in the meta-analysis, with a total of 1537 patients (*n*_experimental group_ = 762; *n*_placebo group_ = 775). There are significant remission differences in ulcerative colitis (relative risk (RR) = 0.81; 95% CI = 0.72–0.91; I^2^ = 32%; *p* = 0.16). However, no significant differences were found in the use of probiotics for the prevention of ulcerative colitis, and for the remission of Crohn’s disease; (4) Conclusions: There are data showing an additional beneficial effect of probiotics on active ulcerative colitis. More and better studies are needed which assess its possible therapeutic efficacy for quiescent ulcerative colitis and for Crohn’s disease.

## 1. Introduction

Inflammatory bowel diseases (IBDs) are a group of auto-immune pathologies, with Crohn’s disease (CD) and ulcerative colitis (UC) among the most well-known. They are both of recurrent clinical course and show a higher incidence in developed countries [1,2].

The etiology of IBDs is unknown—an inappropriate activation is produced of the immune system against the intestinal mucosa in individuals with a genetic predisposition. Environmental factors have been involved, with possible mediators including geo-localization, tobacco, rest disorders, surgical interventions or infections during childhood, and diet—the latter considered a higher risk factor in the development of such pathologies [2,3].

Treatment is focused on the accepted pathogenic mechanisms. The following have been recommended: quitting tobacco use, specific diet and nutrition, anti-inflammatories such as corticoids (temporary use), immuno-suppressors, biological therapy, and surgery [4]. In similar conditions, different types of diet approaches have been developed for IBDs, such as prebiotics/probiotics/postbiotics, to explore their efficacy as possible change agents of the existent inflammatory processes in these diseases [5]. The microbiota is reciprocally related to the human being: it provides various positive effects in the immune system, competing with harmful micro-organisms, or complementing nutrients which are necessary for the human body [6].

The different types are the following: prebiotics, probiotics, or more recently, postbiotics [7]. Prebiotics are food substances that the organism does not digest and which, once they reach the colon, favor the growth of bacterial species of the microbiota considered to produce beneficial effects for the human organism. Among the main benefits are improvement in intestinal motility, both in terms of frequency and of volume; reduction in LDL cholesterol and blood triglycerides; contribution to the synthesis of folic acid; improvement in the immunological function; and, thanks to butyric acid, a reduction in the risk of malignant tumors [8].

The change in the microbiota produced by prebiotics in patients with IBDs, that is, those where low levels of *Firmicutes* and *Bifidobacteria* and a high number of bacteria which are pro-inflammatory for the organism (*Escherichia, Fusobacterium*) have been detected, could favor their remission or maintenance [6,7,9].

Prebiotics are found in foods rich in fiber such as whole grains (wheat, oats, and barley derivatives), vegetables (onion, garlic, leek), fruits (apple, banana), and legumes (soya) [8]. Probiotics are live micro-organisms which have demonstrated a synbiotic effect with the host. Their beneficial effects include re-establishing deficient digestion (sugar intolerances), restitution of the microbiota (diarrhea caused by antibiotics), and prevention of mastitis [8]. Postbiotics are products or a fraction of the lysis of bacteria [10,11]. One of the most studied postbiotics is butyrate, the energetic base of the colonocytes that has suppressive effects on pro-inflammatory cytokines of the intestine [7].

There are studies which proposed treating UC with foods from germinated barley, a butyrate precursor. These studies turned out to be very promising and guided research in this direction [12]. Another study proposed that the *Escherichia coli Nissle 1917* probiotic was as valid as the usual UC treatment with *Mesalazine (5-ASA)* to maintain remission. A few years later, studies conducted with the *De Simone Formulation (DSF)* probiotic (a combination of diverse probiotics) and with *Lactobacillus GG* claimed identical results [13,14]. Likewise, an Italian study found that adding butyric acid in patients with a deficient response to treatment with *Mesalazine* showed good results in maintaining remission in patients with moderate UC [15]. Finally, a compound made of fermented oats, *Lactobacillus plantarum (L. Plantarum) 299v*, barley malt, lecithin, and water (*Profermín*) was tested, which performed well for remission patients in UC [16].

The latest studies have been focusing fundamentally on the use of probiotics and postbiotics. Recent studies have noted that Crohn’s disease and UC affect 1.4 million people in the United States, 2.5 million people in Europe, and millions more in the rest of the world. Besides, during the second half of the 21st century, the incidence of these diseases has risen in developed countries, reaching numbers of 6–10/100,000 inhabitants/year in Europe [8]. These figures apply to Spain, both in terms of the increasing number of cases and in the cardinal differences, and are evident in the regions of Córdoba and Seville [1].

Considering the chronicity and development of outbreaks of these diseases, and their predominant onset between childhood and the first half of life, it can be asserted that the treatment of IBDs consumes a large quantity of resources, both economic and personal, as well as for the health system (diagnoses tests, drugs, hospital stays, health personnel, etc.) and the work environment due to the length of possible sick leave [17]. As an example, the cost of caring for a patient with Crohn’s disease is estimated at approximately EUR 7000 per year [17,18], 57% of which is attributable to hospital admissions, 33% to pharmaceutical expenses, and the remainder to different causes such as consultations, tests, and surgical interventions [18].

As a consequence of this, the following research question arose: In individuals with inflammatory bowel disease (P), is the use of probiotics (with diverse families and strains) (I) efficient for starting/maintaining the remission of these diseases (O) with respect to the use of the placebo or conventional treatment (C)?

In order to answer that question, the following objective was set out: to analyze the efficacy of using probiotics in patients with inflammatory bowel disease in the active or quiescent phase as a coadjuvant therapy at the beginning of remission or maintenance.

## 2. Methods

### 2.1. Search Strategy and Inclusion Criteria

A bibliographic search was performed in scientific databases and sources including Medline, PubMed, EMBASE, Scopus, Web of Science, and The Cochrane Library, according to the recommendations set forth in Preferred Reporting Items for Systematic Reviews and Meta-Analyses for Protocols (PRISMA), and was evaluated with the AMSTAR-2 tool (Table S1) [19,20]. Additionally, a search was performed in the Clinical Trial Gov record page, and the nonexistence of similar papers was checked against the Prospero record. The protocol of this study was registered in the Prospero website in February 2019 (ID: 168533; status: not yet registered; last edited: 9 February 2020; consulted: 23 April 2020).

The search terms of (“inflammatory bowel diseases” OR “crohn disease” OR “colitis, ulcerative” OR “ulcerative colitis” OR ileitis OR colitis) and (prebiotics OR probiotics OR Saccharomyces OR Lactobacillus OR Bifidobacterium OR “escherichia coli”) and their equivalent in Spanish were used. The descriptors of the search equation were chosen from the Medical Subject Headings (MeSH) thesaurus.

The search was performed from January to December 2019. The inclusion criteria were articles published in the last 10 years, in English/Spanish, with a theme related to the objectives of this study, and randomized clinical trials (RCTs) performed on adult human beings. Children were not included in the difference in diagnostic criteria, a narrower therapeutic margin and limitation in the use of probiotics for the treatment of these diseases. Only RCTs were selected to increase the methodological quality of the review and to reduce the number of biases on a theme which, by its very nature, is complex to approach due to the great number of probiotic families, dosing, and stage of the inflammatory bowel diseases.

### 2.2. Data Extraction 

Ethical approval was not necessary because of the type of study (systematic review with meta-analysis) and because no patients were involved. The search and selection of articles were performed independently by two researchers; an expert in inflammatory bowel diseases was consulted for resolution in the case of disagreement. Titles and abstracts were read first, then the selected article in its entirety. A reverse and forward bibliographic search was performed of the cited references in the selected studies. The agreement degree between the two researchers in terms of assessing the eligibility of the study was measured using Kappa’s statistical test.

A data coding handbook was used. The following variables were obtained from each study: (1) author; (2) year of publication; (3) country of origin; (4) study design; (5) sampling size; (6) criteria used to define remission; (7) types of probiotics of the experimental group and duration; (8) control used and duration; (9) results. Data were extracted as an intention-to-treat analysis.

The primary dichotomous results evaluated were the efficacy of the probiotics, comparing it with the usual treatment (*Mesalazine or 5-ASA*) or with the placebo in terms of failure to achieve remission in active IBDs and relapse in quiescent IBDs.

### 2.3. Quality and Bias Risk Assessment

The bias risk assessment tool was used according to Cochrane [21], which classifies each type of risk as low, high, or unclear. The types of risk included the following: generation of random sequences, allocation concealment, blinding of the participants and of the personnel, blinding of the results evaluation, data from incomplete results, selective reports, and other possible bias sources. It was considered that the studies without a high bias risk in any category were of high quality (1++) and that those with one high risk or two unclear risks were of medium quality (1+). The rest were considered of low quality (1−). 

The statistical analysis and the bias evaluation were performed with the Review Manager software, version 5.3 (Cochrane Library, London, UK). Data were also imported into the Grade Pro application, which allows a valuation to be given to the recommendation degree of the data obtained.

### 2.4. Data Synthesis and Statistical Analysis

Relative risk (RR) was used to compare dichotomous variables with a 95% confidence interval (CI). The continuous variables were evaluated through weighted mean differences (WMDs) with a 95% CI. When the study did not offer data on the standard deviation, the Hozo et al. [22] method was used. 

Both the binary and continuous data were calculated using random and fixed effects models. The fixed effects model was initially selected if there was not high heterogeneity among the studies (I2 ≤ 50); otherwise, the random effects model was used [23]. 

The heterogeneity among the studies was evaluated by means of a chi-square test and I^2^ test, with a statistical significance level of *p* < 0.05. I^2^ values between 0% and 25% suggested low heterogeneity, over 25% moderate heterogeneity, and over 75% high heterogeneity [24]. 

A forest plot was used for the graphical representation of the meta-analysis results, and a funnel plot was used to evaluate the publication bias among the studies. The asymmetry of this last graphic was assessed by means of the funnel representation and with a statistical significance level of *p* < 0.05 by the Eggers test, which indicates evidence of publication bias. 

The subgroup analysis was performed based on the IBD typology (UC or Crohn’s disease), whether in the active or quiescent phase. A sensitivity analysis was performed to assess the soundness of the results by serial omission of each study. A *p*-value < 0.05 was considered statistically significant.

Data from dichotomous results were grouped by means of a random effects model [23] to offer a more conservative estimation of the effects of the probiotics on the IBDs, which allows for heterogeneity among studies. The impact of the probiotics, compared to the usual treatment (*Mesalazine, 5-ASA*, etc.) or placebo, was expressed as an RR of not achieving remission with 95% confidence intervals (CIs) in the therapy trials for active UC or Crohn’s disease, or as RR of relapse in therapy trials for quiescent UC.

## 3. Results

### 3.1. Results Obtained in the Selection of Articles

**Figure 1:**
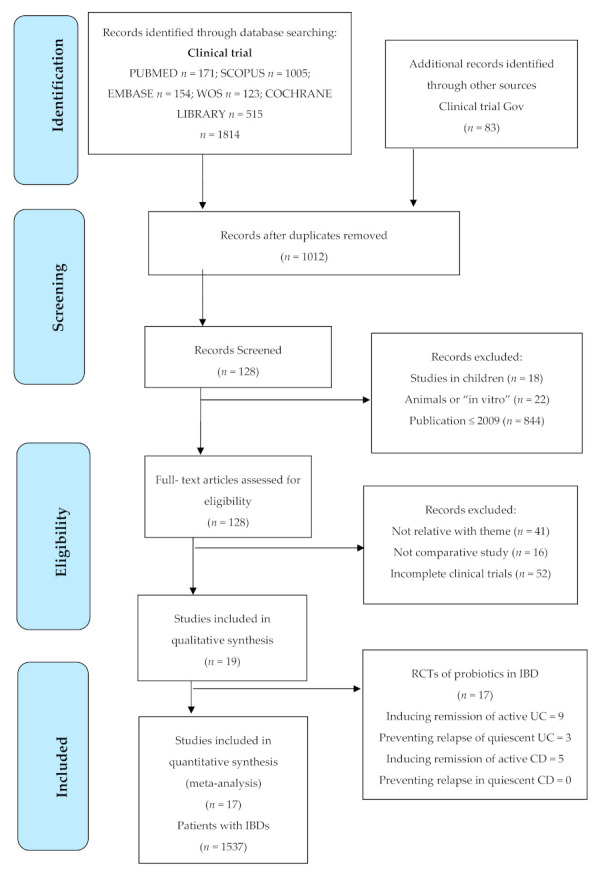
Flow diagram in the selection of articles according to PRISMA. CD = Crohn’s disease; IBDs = inflammatory bowel diseases; UC = ulcerative colitis.

A total of 1814 articles were identified in the initial literature search, and 83 additional documents that were found in clinical trial specific records were excluded (Clinical Trial Gov and Prospero). After deleting 885 duplicate articles by means of the Mendeley^®^ bibliographic manager and applying the inclusion criteria and assessing the articles’ titles and abstracts, 1878 were excluded for not meeting the inclusion criteria. Finally, 19 studies were selected in the analysis for the systematic review, of which 17 offered data to perform the meta-analysis, spanning a sample of 1537 participants with IBDs (experimental group, *n* = 762; placebo group, *n* = 775), as shown in Figure 1. The agreement among the researchers with regards to the assessment of the eligibility of the trials was excellent (Kappa statistics = 0.91).

### 3.2. Descriptive Analysis of the Results Found

Of the 19 clinical trials included in the systematic review, 36.84% (*n* = 7) were randomized, and 63.15% (*n* = 12) were double-blind randomized; no crossed trials were found. Of these, four were published in 2010; three were published in 2019 and 2011; two were published in 2018, 2015, and 2009; and one was published in 2013, 2014, and 2016. The evidence levels evaluated according to the quality of the selected articles were given a score of 1++ in 26.31% (*n* = 5) of cases, the same percentage was scored 1+ (*n* = 5), and the remaining 47.36% (*n* = 9) was scored 1−. 

The probiotics studied belonged to different families and were administered with different doses. Four studies used a synbiotic which is the combination of a probiotic with a prebiotic. In relation to the families used, of a total of 36 different possible agents, 63.15% (*n* = 12) used *Lactobacillus*; 57.89% (*n* = 11) used a strain of the Bifidobacterium family; 21.05% (*n* = 4) used the *DSF* compound; 10.52% (*n* = 2) used *Escherichia coli*, *Enterococcus*, and *Streptococcus*; and 5.26% (*n* = 1) were composed of *Clostridium*, *Bacillus mesentericus*, and *Saccharomyces boulardii*, taking into account that there are articles in which diverse probiotic families were analyzed. 

The mean duration of the treatments was 4.6 months, the longest being 12 months in 26.31% (*n* = 5) of the studies, and the shortest 0.5 months in 5.26% (*n* = 1) of cases.

The details of each article included are provided in Table S2.

### 3.3. Bias Risk Assessment of the Selected Studies and Publication Bias

**Figure 2:**
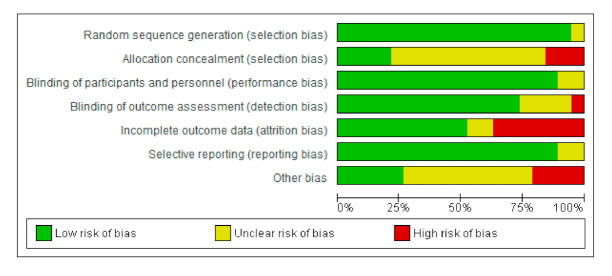
Bias analysis of the articles selected altogether.

**Figure 3:**
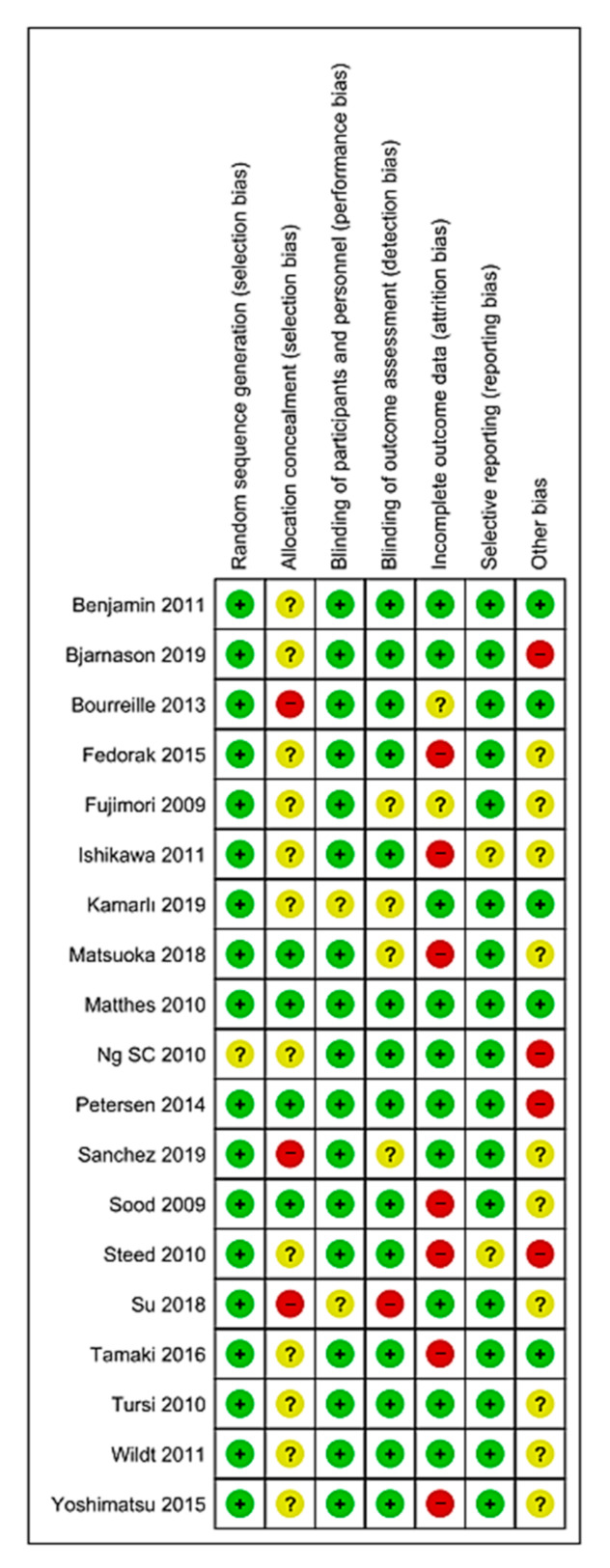
Bias analysis of the articles selected one by one. Red = high risk; Green = low risk; Yellow/? = unclear risk; +/− = risk percentage.

The bias risk was assessed using RevMan 5^®^, represented in Figure 2 and Figure 3 by means of bias assessment graphics of all the studies included (Figure 2) and through a summary one-by-one graphic (Figure 3). Allocation concealment was evidenced in 25%, with approximately an 80% blinding of the participants and of the personnel, 75% blinding of the results assessment, and with a generation of a random sequence below 90%.

**Figure 4:**
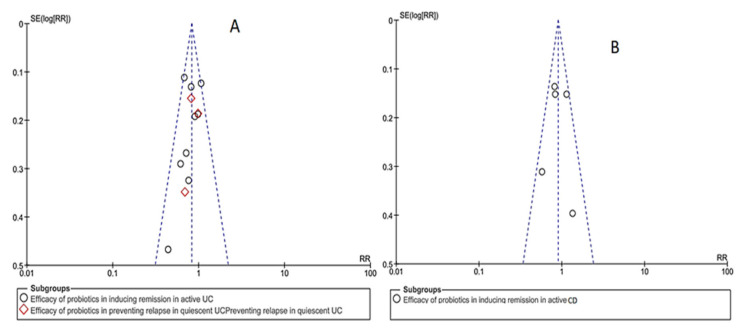
Analysis of publication bias by means of a funnel plot. (**A**): ulcerative colitis publications (**B**): Crohn’s disease publications. CD = Crohn’s disease; RR = relative risk; SE = publication bias; UC = ulcerative colitis.

In relation to the publication bias, in a funnel plot for each objective of the study assessed, an inverted funnel can be observed, the most powerful studies being concentrated in the center (Figure 4).

### 3.4. Results of the Meta-Analysis

Of the 17 studies included in the meta-analysis, 52.94% (*n* = 9) of the RCTs assessed the efficacy of the probiotics to induce remission of active UC, 17.64% (*n* = 3) studied their efficacy in preventing relapse in quiescent UC, 29.41% (*n* = 5) analyzed their efficacy in inducing remission in active Crohn’s disease, and no studies were found that assessed the efficacy of probiotics in preventing relapse in quiescent Crohn’s disease. The permitted and excluded medications in each of these studies are described in Table S3. 

### 3.5. Efficacy of the Probiotics to Induce Remission in Active UC

One of the nine trials, with a sample of 120 patients, compared probiotics with prebiotics and synbiotics for the induction of the remission of active UC [25], and the remaining eight trials were placebo-controlled [26,27,28,29,30,31,32,33]. Two trials presented a low bias risk [29,30]. Improvements were achieved in the assessment of the IBDs in the three groups, the probiotic group improving the emotional function (*p* = 0.03), the prebiotic group improving the intestinal function (*p* = 0.04), and the synbiotic group improving the social realm and the systematic function (*p* = 0.008 and *p* = 0.002, respectively). 

The seven RCTs which were placebo-controlled contained a total of 639 patients with active UC [26,27,28,29,30,31,32,33]. In total, 201 out of 362 (55.52%) patients allocated to probiotics did not achieve remission, versus 271 out of 397 (68.26%) patients in the placebo group (RR to achieve remission = 0.81; 95% CI = 0.72–0.91), with heterogeneity among the studies (I^2^ = 32%, *p* = 0.16). 

Three of the trials used the probiotic formulation previously known as VSL#3. The probiotic formulation that was assessed in these studies is now known by the generic name of *De Simone Formulation (DSF)*, available under the brand name Vivomixx in the EU [26,27,28,29]. When only these three studies were considered in the analysis, 88 out of 162 (56.2%) patients randomly allocated to *DSF* did not achieve remission, versus 118 out of 157 (75.2%) in the placebo group (RR to achieve remission = 0.72; 95% CI = 0.61–0.85), with no heterogeneity among the studies (I^2^ = 16%, *p* = 0.30). 

Two RCTs used *E. Coli* [27,30] in a sample of 164 patients. In those studies, 56 out of 95 (58.9%) patients allocated active therapy did not achieve remission, versus 59 out of 95 (62.1%) allocated to the placebo (RR = 0.94; 95% CI = 0.72–1.23), with no heterogeneity among the studies (I^2^ = 0%, *p* = 0.76). 

The rest of the studies used different families, especially *Lactobacillus* and *Bifidobacterium*, among others [25,31,33]. Of the patients, 57 out of 105 (54.2%) allocated to active therapy did not achieve remission, versus 94 out of 145 (64.82%) allocated to the placebo (RR = 0.88; 95% CI = 0.72–1.07), with no heterogeneity among the studies (I^2^ = 39%, *p* = 0.18).

If we evaluate by strains, an improvement was observed in those studies that used a combination of microorganism (mixture) versus a single strain in patients with UC (Figure S1) and CD (Figure S2).

### 3.6. Efficacy of Probiotics to Prevent Relapse in UC

There were three placebo-controlled trials [34,35,36] with a total sample of 284 patients which reported the efficacy of the probiotics versus the placebo in preventing clinical or endoscopical relapse of UC. In total, 60 out of 147 (55.52%) patients allocated to probiotics did not achieve remission, versus 60 out of 137 (68.26%) allocated to the placebo (RR of failure to achieve prevention = 0.89; 95% CI = 0.68–1.15), with heterogeneity among the studies (I^2^ = 0%, *p* = 0.59). 

In relation to the families used, two of them employed the *Bifidobacterium*–*Lactobacillus acidophilus* [35,37] combination, whereas the other used a compound of 2 mg *Streptococcus faecalis* T–110, 10 mg *Clostridium butyricum TO–A*, and 10 mg *Bacillus mesentericus TO–A* [38].

**Figure 5:**
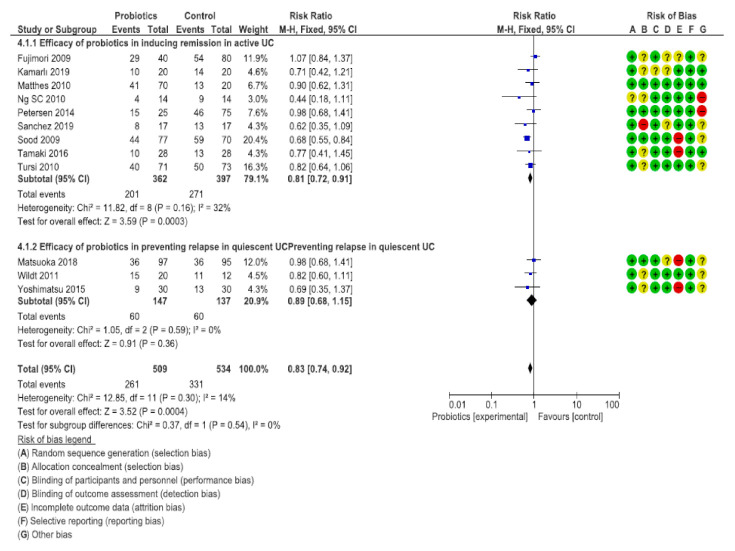
Comparison of probiotics versus placebo groups for the remission and prevention of ulcerative colitis (UC). CD = Crohn’s disease; Chi^2^ = test Chi^2^; CI = confidence interval; Green = low risk; M-H = Mantel-Haenszel method; P = statistical significance; Red = high risk; UC = ulcerative colitis; Yellow/? = unclear risk; Z = test for overall effect; +/− = risk percentage.

In relation to the general assessment in UC, with a total of 1043 participants, 261 out of 509 (51.27%) patients allocated to probiotics did not achieve remission, versus 331 out of 534 (61.98%) allocated to the placebo (RR = 0.83; 95% CI = 0.74–0.92), with heterogeneity among the studies (I^2^ = 14%, *p* = 0.30), as shown in Figure 5 and Table 1. A combination of different strains of probiotics were used.

### 3.7. Efficacy of the Probiotics for the Remission of Crohn’s Disease

There were five placebo-controlled trials [39,40,41,42,43] with a sample of 494 patients which reported the efficacy of the probiotics versus the placebo in terms of clinical or endoscopical remission of Crohn’s disease.

In total, 126 out of 253 (49.80%) patients allocated to probiotics did not achieve remission, versus 134 out of 241 (55.60%) allocated to the placebo (RR of failure to achieve remission = 0.90; 95% CI= 0.77–1.06), with heterogeneity among the studies (I^2^ = 37%, *p* = 0.18). 

**Figure 6:**
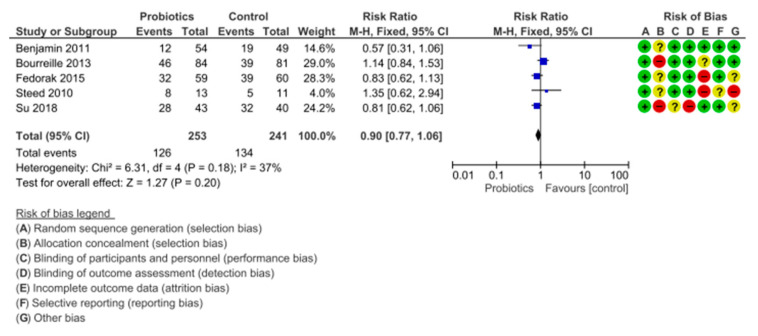
Comparison of probiotics versus placebo groups for the remission of Crohn’s disease. CD = Crohn’s disease; Chi^2^ = test Chi^2^; CI = confidence interval; Green = low risk; M-H = Mantel-Haenszel method; P = statistical significance; Red = high risk; UC = ulcerative colitis; Yellow/? = unclear risk; Z = test for overall effect; +/− = risk percentage.

In relation to the families used, there was great diversity among studies, since each of them used a different family (*Bifidobacterium* [39,43], *Saccharomyces boulardii* [41], *DSF* [42]) and one of the studies even used a prebiotic (*fructo-oligosaccharides*) to assess remission [40], as shown in Figure 6 and Table 2. 

## 4. Discussion 

The objective of this systematic review with meta-analysis was to analyze the efficacy of using probiotics in patients with IBDs as a coadjuvant therapy at the beginning of remission or after that period. We performed an exhaustive analysis of both groups of IBDs (UC and Crohn’s disease) and compared the effectiveness of the probiotics both in the outbreaks and in the remissions of those diseases.

Most studies analyzed the efficacy of the probiotics in active UC, as shown in Table S4, with a statistically significant *p* value found in the majority. This meta-analysis does not corroborate the previous findings on their efficacy in quiescent UC and Crohn’s disease; this may be due to the fact that the researchers who carried out these studies assessed effectiveness only partially and used different criteria to assess remission. 

### 4.1. Efficacy of the Probiotics in UC 

A 2017 meta-analysis by Derwa et al. [44] shows a certain discrepancy to the results of this study, where the probiotics had a beneficial effect on the remission of UC (RR to achieve remission = 0.81; 95% CI = 0.72–0.91). Here, after analyzing 22 RCTs, no benefits were found with the probiotics over the placebo in terms of inducing remission in active UC (RR to achieve remission = 0.86; 95% CI = 0.68–1.08). 

Fujiya et al. [45] documented the same findings in their meta-analysis of 20 RCTs published in 2014, with the following results: RR = 1.81; 95% = 1.40–2.35. However, both studies highlight the variability of the conditions in these studies, which generates the need for an additional analysis that evaluates the effects of probiotic treatments in IBDs to try to clarify their effectiveness and an optimum treatment regime for each condition or population of these patients.

However, the findings of Derwa et al. [44] are similar to those presented in this study when performing the analysis by subgroups, since it was only when the *DSF* trials were considered that the authors found a benefit (RR = 0.74; 95% CI = 0.63–0.87) in accordance with the results of this meta-analysis (RR = 0.72; 95% CI = 0.61–0.85), which is the reason why they concluded that *DSF* can be efficient to induce remission in active UC. 

The results found by Jia et al. [46] go along the same line—they did not observe significant differences in the remission, relapse, and complication rates in the general results of a meta-analysis between probiotics and the placebo group, but the subgroup analyses suggested that *DSF* had a higher remission rate and a lower relapse rate (RR = 1.67, 95% CI = 1.06–2.63, *p* = 0.03; RR = 0.29, 95% CI = 0.10–0.83, *p* = 0.02).

However, the meta-analysis performed by Ganji-Arjenaki et al. [47] in 2018 shows similar results to those found in this study in relation to probiotics and UC. The authors described that the analysis of three trials with IBDs revealed a significant advantage of the probiotics (*p* < 0.01), and the analysis of 18 clinical trials revealed that the probiotics had a significant effect (*p* = 0.007) in patients with UC in different conditions. The study also reasserted that *DSF* had a significant effect (*p* < 0.01) on patients with UC. 

In accordance with these results are those presented by Shen et al. [48] in a meta-analysis of 23 RCTs with a total of 1763 participants, where they stated that the probiotics significantly increased the remission rates in patients with active UC (*p* ≤ 0.001). Additionally, the subgroup analysis found that *DSF* significantly increased remission rates when compared with the controls in patients with active UC (*p* = 0.004). 

Along the same line, the study conducted by Dong et al. [49] suggested that probiotics have potential benefits for patients with UC.

The effectiveness of *DSF* probiotics is also supported by the 2014 meta-analysis by Mardini et al. [50] performed on three low-risk studies with a total of 319 patients. They documented a 50% reduction in the activity index of the UC disease in 44.6% of the patients treated with *DSF* versus 25.1% of the patients who received the placebo (*p* = 0.008; OR = 2.793; 95% CI = 1.375–5.676), and the remission rate was 43.8% in patients treated with *DSF* versus 24.8% in patients who received the placebo (*p* = 0.007; OR = 2.4; 95% CI = 1.48–3.88). These results support the findings of this study in relation to the use of *DSF*. 

In 2011, Ishikawa et al. [40] assessed the use of synbiotics for the remission of active UC. A significant reduction was obtained in *Bacteroidaceae* and *Mieloperoxidasa*. The authors concluded that using *B. Breve Yakult* strain and *GOS* can improve the clinical condition of the patients with UC.

Along that same line, recent randomized clinical trials such as that conducted by Yoshimatsu et al. [36] with a total sample of 46 patients in which they used a combination of *Streptococcus faecalis T-110*, 10 mg *Clostridium butyricum TO-A*, and 10 mg *Bacillus mesentericus TO-A* as a probiotic therapy support the findings of this study in relation to the use of probiotics in UC. The authors obtained the following relapse rates in the probiotics and placebo groups, respectively: 0.0% versus 17.4% at 3 months (*p* = 0.036), 8.7% versus 26.1% at 6 months (*p* = 0.119), and 21.7% versus 34.8% at 9 months (*p* = 0.326). At 12 months, the remission rates were 69.5% in the probiotics group and 56.6% in the placebo group (*p* = 0.248). In view of that, they also concluded that probiotics can be effective to maintain clinical remission in patients with quiescent UC. 

However, a *B-FLORA* double-blind study conducted by Matsuoka et al. [35] with a total of 195 patients with quiescent UC, randomized to receive a pack of *Bifidobacterium Breve* fermented milk per day of Bifidobacterium breve Yakult strain and *Lactobacillus acidophilus* for 48 weeks, did not obtain significant differences in relapse rates between the BFM and placebo groups (*p* = 0.643), which led to the discontinuation of the study due to lack of efficacy. The different findings reveal the discrepancy in the probiotic strains used, as well as in the treatment dose and duration. In relation to our study, these last authors found similar results to those obtained in this meta-analysis; that is, no clear benefit was evidenced in the experimental group versus the control group.

On the other hand, several studies show that mixed probiotics are more effective than using individual probiotics. The analysis of these clinical trials showed a significant benefit of probiotic use, especially when the strains were mixed [26,28,29,32,33]. These findings were previously demonstrated in pouchitis with the use of *DSF* [48,51,52]. The study conducted by Ganji-Arjenaki et al. [47] shows similar results to our study (Figure S1). These authors concluded that mixed probiotics in patients with UC had a significant effect (*p* < 0.01), and the combination of *Lactobacillus* and prebiotics had a significant effect (*p* = 0.03) only in patients with UC.

### 4.2. Efficacy of Probiotics in Crohn’s Disease

In relation to the efficacy of probiotics for the remission of Crohn’s disease, the data in this meta-analysis show an RR of failure to achieve remission of 0.90 (95% CI = 0.77–1.06) in the probiotics group versus the placebo group. This result coincides with the data offered by the study of Shen et al. [48], where the probiotics did not obtain any significant benefit in the subgroup of patients with Crohn’s disease (*p* = 0.35, RR = 0.89). 

Derwa et al. [44] obtained similar results from the analysis they conducted with two RCTs with a total of 37 participants, where they found an RR of failure to achieve remission of 0.99 (95% CI = 0.57–1.72). 

In accordance with the aforementioned literature, the results obtained by Ganji-Arjenaki et al. [47] do not evidence any significant effect on Crohn’s disease from using probiotics versus the control group (*p* = 0.07). 

The RCT conducted by Fedorak et al. [30] found significant effects of using probiotics for the remission of Crohn’s disease only when treatment exceeded 90 days. Their results showed that the patients who received *DSF* had reduced levels of inflammatory cytokines of the mucosa in comparison with the placebo at day 90 (*p* < 0.05). However, they did not find any significant differences in the rates of endoscopic recurrence, which is the reason why they concluded that this probiotic must be further investigated in its role to prevent the recurrence of Crohn’s disease.

The results of the double-blind RCT published in 2019 by Bjarnason et al. [53], conducted with 81 and 61 patients with UC and Crohn’s disease, respectively, were in accordance with the findings of this meta-analysis. They did not obtain any statistical significance in the probiotic group versus the control group in Crohn’s disease (Figure S2); however, their results were more promising in relation to UC, nearing significance (*p* = 0.076), and the post hoc analyses showed that levels of faecal calprotectin were significantly reduced (*p* < 0.015) in patients with UC who received the probiotic instead of the placebo. Regarding the analysis of probiotic mixtures compared to those used alone, there is only one study that has used mixed probiotics, and this is the most promising study in terms of efficacy [30].

### 4.3. Limitations

Despite the evidence of low heterogeneity among the studies included in the meta-analysis performed, a certain variability was observed in the treatments in terms of strains, doses, and duration, and many studies did not compare patients with the same degree of severity and used different criteria to define the remission of disease. Other limitations include the limited evidence provided for the use of any probiotic in the management of IBDs, but this is one of the reasons for carrying out this meta-analysis. To ensure high methodological quality, one of the inclusion criteria chosen was to include clinical trials only. Although the meta-analysis is at the peak of the evidence pyramid, it is true that the conclusions drawn from these studies must take into account these limitations and the possible biases and influence of certain moderating variables.

## 5. Conclusions

The use of probiotics and prebiotics appears to have greater clinical relevance in the treatment of UC than CD, being more significant in the remission of active UC than quiescent UC. In addition, the use of a mixture of probiotics appears to be superior to using a simple strain to induce UC remission.

In relation to preventing quiescent UC, more studies are needed to determine if the regulated use of probiotics can reduce relapse rates. Considering the wide array of genotypes and phenotypes of this condition, it is quite conceivable that the studies performed to date have not yet identified the specific probiotics, or the specific dosages, that may be beneficial in the various forms of this inflammatory process. 

On the other hand, no evidence of clinical significance in the management of Crohn’s disease has been found. The latest studies have focused on the use of probiotics and postbiotics and the regulation of microbiota. Despite this, more research that is not sponsored by the probiotic industry is needed on this regarding how the microbiota is modified in these cases and the effects on patients diagnosed with IBDs. To deepen our understanding in relation to this theme, it is necessary to unify the criteria for the duration of treatments, manage more relevant strains, and use identical criteria for clinical remission. 

In conclusion, the complementary use of probiotics might be recommended in patients with UC, although homogeneous studies are still needed to assess the effectiveness of these in patients with IBDs, especially in relation to Crohn’s disease. Future studies should be guided in the combined use of probiotics strains, as it appears to show greater clinical efficacy. This is how the dysbiosis of patients with IBDs would be balanced.

The implications for the clinical practice of the results of this research study suggest an advancement in the health and quality of life of patients with IBDs, as well as the promotion of advancements in the treatment of these diseases, which constitute a public health problem affecting millions of patients and that have enormous repercussions at sanitary, working, and social levels, with a great impact on the socio-sanitary costs of each country worldwide. 

## Figures and Tables

**Table 1:** Recommendation degree of probiotic versus placebo groups for the remission and prevention of UC.

Probiotic Compared with Placebo for Ulcerative Colitis					
Outcome	No. of Participants (Studies) Follow-Up	Certainty of the Evidence (GRADE)	Relative Effect (95% CI)	Absolute Anticipated Effects	
				Placebo Risk	The Risk Difference with Probiotics
Efficacy of probiotics in inducing remission in active UC	759 (9 RCTs)	⨁⨁⨁◯ MODERATE	RR 0.81 (0.72 to 0.91)	683 × 1000	130 less to 1000 (191 less to 61)
Efficacy of probiotics in preventing relapse in quiescent UC	284 (3 RCTs)	⨁⨁⨁◯ MODERATE	RR 0.89 (0.68 to 1.15)	438 × 1000	48 less to 1000 (140 less to 66)

Patient or Population: Ulcerative Colitis, Outcome: Efficacy, Intervention: Probiotic, Comparison: Placebo. The risk in the intervention group (and its 95% confidence interval) is based on the risk assumed in the comparison group and the relative effect of the intervention (and its 95% confidence interval). CI: Confidence Interval; RR: Risk Ratio. GRADE Working Group grades of evidence. High certainty: We are very confident that the true effect lies close to that of the effect estimate. Moderate certainty: We are moderately confident in the effect estimate—the true effect is likely to be close to the effect estimate, but there is a possibility that it is substantially different. Low certainty: Our confidence in the effect estimate is limited—the true effect may be substantially different from the effect estimate. Very low certainty: We have very little confidence in the effect estimate—the true effect is likely to be substantially different from the effect estimate.CI = confidence interval; UC = ulcerative colitis; RCTs = randomized controlled trial; RR = relative risk; ⨁⨁⨁◯ = level of recommendation.

**Table 2:** Recommendation degree of probiotics versus placebo for the remission of Crohn’s disease (CD).

Probiotic Compared with Placebo for Crohn’s Disease					
Outcome	No. of Participants (Studies) Follow-Up	Certainty of the Evidence (GRADE)	Relative Effect (95% CI)	Absolute Anticipated Effects	
				Placebo Risk	The Risk Difference with Probiotics
Efficacy of probiotics in inducing remission in active Crohn’s disease	494 (5 RCTs)	⨁⨁⨁◯ MODERATE	RR 0.90 (0.77 to 1.06)	556 × 1000	56 less to 1000 (128 less to 33)

Patient or population: Crohn’s disease, Outcome: Efficacy, Intervention: Probiotic, Comparison: Placebo. The risk in the intervention group (and its 95% confidence interval) is based on the risk assumed in the comparison group and the relative effect of the intervention (and its 95% confidence interval). CI: Confidence Interval; RR: Risk Ratio. GRADE Working Group grades of evidence. High certainty: We are very confident that the true effect lies close to that of the effect estimate. Moderate certainty: We are moderately confident in the effect estimate—the true effect is likely to be close to the effect estimate, but there is a possibility that it is substantially different. Low certainty: Our confidence in the effect estimate is limited—the true effect may be substantially different from the effect estimate. Very low certainty: We have very little confidence in the effect estimate—the true effect is likely to be substantially different from the effect estimate.CI = confidence interval; RCTs = randomized controlled trial; RR = relative risk; ⨁⨁⨁◯ = level of recommendation.

## Supplementary Materials

The following are available online at https://www.mdpi.com/2072-6643/12/9/2628/s1, Figure S1: Single species versus mixture for the remission of UC, Figure S2: Single species versus mixture for the remission of CD, Table S1: AMSTAR 2: a critical appraisal tool for systematic reviews that include randomized or nonrandomized studies of healthcare interventions, or both, Table S2: Results of the articles selected in the systematic review, Table S3: Concomitant Medication in Included Studies, Table S4: Outcomes of randomized controlled trials evaluating the effects of probiotics on IBDs.
