# Supplementary material for: Probiotics as a Coadjuvant Factor in Active or Quiescent Inflammatory Bowel Disease of Adults—A Meta-Analytical Study
Source: Nutrients. 2020 Aug 28;12(9):2628. doi: 10.3390/nu12092628 (PMC7551006; doi:10.3390/nu12092628)
Supplement: Supplementary file 1 [file nutrients-12-02628-s001.pdf]

SUPPLEMENTARY MATERIAL

Table S1. AMSTAR 2: a critical appraisal tool for systematic reviews that include randomized or nonrandomized studies of healthcare interventions, or both

**1. Did the research questions and inclusion criteria for the review include the components of PICO?**		
For Yes:<br>X Population<br>X Intervention<br>X Comparator group<br>X Outcome	Optional (recommended)<br>Timeframe for follow-up	**Yes**<br>No
**2. Did the report of the review contain an explicit statement that the review methods were established prior to the conduct of the review and did the report justify any significant deviations from the protocol?**		
For Partial Yes:<br>The authors state that they had a written protocol or guide that included ALL the following:<br>X review question(s)<br>X a search strategy<br>X inclusion/exclusion criteria<br>X a risk of bias assessment	For Yes:<br>As for partial yes, plus the protocol should be registered and should also have specified:<br>X a meta-analysis/synthesis plan, if appropriate, *and*<br>X a plan for investigating causes of heterogeneity<br>X justification for any deviations from the protocol	**Yes** Partial Yes No
**3. Did the review authors explain their selection of the study designs for inclusion in the review?**		
For Yes, the review should satisfy ONE of the following:<br>*X Explanation for* including only RCTs<br>☐ OR *Explanation for* including only NRSI<br>☐ OR *Explanation for* including both RCTs and NRSI		**Yes**<br>No
**4. Did the review authors use a comprehensive literature search strategy?**		
For Partial Yes (all the following):	For Yes, should also have (all the	

X searched at least 2 databases (relevant to research question)<br>X provided key word and/or search strategy<br>X justified publication restrictions<br>(e.g. language)	following):<br>X searched the reference lists / bibliographies of included studies<br>X searched trial/study registries<br>X included/consulted content experts in the field<br>X where relevant, searched for grey literature<br>conducted search within 24 months of completion of the review	Yes Partial Yes No

5. Did the review authors perform study selection in duplicate?

For Yes, either ONE of the following:<br>X at least two reviewers independently agreed on selection of eligible studies and achieved consensus on which studies to include<br>☐ OR two reviewers selected a sample of eligible studies and achieved good agreement (at least 80 percent), with the remainder selected by one reviewer.	**Yes**<br>No

6. Did the review authors perform data extraction in duplicate?

For Yes, either ONE of the following:<br>X at least two reviewers achieved consensus on which data to extract from included studies<br>☐ OR two reviewers extracted data from a sample of eligible studies and achieved good agreement (at least 80 percent), with the remainder extracted by one reviewer.	☐ No	**Yes**

7. Did the review authors provide a list of excluded studies and justify the exclusions?

For Partial Yes:<br>provided a list of all potentially relevant studies that were read in full-text form but excluded from the review	For Yes, must also have:<br>Justified the exclusion from the review of each potentially relevant study	Yes<br>☐ Partial Yes<br>☐ No

8. Did the review authors describe the included studies in adequate detail?

For Partial Yes (ALL the following):<br>X described populations<br>X described interventions<br>X described comparators<br>X described outcomes<br>X described research designs	For Yes, should also have ALL the following:<br>X described population in detail<br>☐ described intervention in detail (including doses where relevant)<br>X described comparator in detail (including doses where relevant)<br>X described study's setting<br>X timeframe for follow-up	☐ Yes<br>Partial Yes<br>☐ No

9. Did the review authors use a satisfactory technique for assessing the risk of bias (RoB) in individual studies that were included in the review?

**RCTs**<br>For Partial Yes, must have assessed RoB from<br>X unconcealed allocation, *and*<br>X lack of blinding of patients and assessors when assessing outcomes (unnecessary for objective outcomes such as all-cause mortality)	For Yes, must also have assessed RoB from:<br>X allocation sequence that was not truly random, *and*<br>X selection of the reported result from among multiple measurements or analyses of a specified outcome	Yes<br>☐ Partial Yes<br>☐ No<br>☐ Includes only NRSI

NRSI

For Partial Yes, must have assessed RoB:

- ☐ from confounding, *and*
- ☐ from selection bias

For Yes, must also have assessed RoB:

- ☐ methods used to ascertain exposures and outcomes, *and*
- ☐ selection of the reported result from among multiple measurements or analyses of a specified outcome

- ☐ Yes
- ☐ Partial Yes
- ☐ No
- ☐ Includes only RCTs

10. Did the review authors report o

n the sources of funding for the studies inc

luded in the review?

For Yes

Must have reported on the sources of funding for individual studies included in the review. Note: Reporting that the reviewers looked for this information but it was not reported by study authors also qualifies

Yes

No

11. If meta-analysis was performed did the review authors use appropriate methods for statistical combination of results?

RCTs

For Yes:

X The authors justified combining the data in a meta-analysis

X AND they used an appropriate weighted technique to combine study results and adjusted for heterogeneity if present.

X AND investigated the causes of any heterogeneity

Yes

- ☐ No
- ☐ No meta-analysis conducted

For NRSI

For Yes:

- ☐ The authors justified combining the data in a meta-analysis
  - ☐ AND they used an appropriate weighted technique to combine study results, adjusting for heterogeneity if present
  - ☐ AND they statistically combined effect estimates from NRSI that were adjusted for confounding, rather than combining raw data, or justified combining raw data when adjusted effect estimates were not available
  - ☐ AND they reported separate summary estimates for RCTs and NRSI separately when both were included in the review

- ☐ Yes
- ☐ No
- ☐ No meta-analysis conducted

**12. If meta-analysis was performed, did the review authors assess the potential impact of RoB in individual studies on the results of the meta-analysis or other evidence synthesis?**	
For Yes:<br>☐ included only low risk of bias RCTs<br>X OR, if the pooled estimate was based on RCTs and/or NRSI at variable RoB, the authors performed analyses to investigate possible impact of RoB on summary estimates of effect.	Yes<br>☐ No<br>☐ No meta-analysis conducted
**13. Did the review authors account for RoB in individual studies when interpreting/ discussing the results of the review?**	
For Yes:<br>☐ included only low risk of bias RCTs<br>X OR, if RCTs with moderate or high RoB, or NRSI were included the review provided a discussion of the likely impact of RoB on the results	Yes<br>☐ No
**14. Did the review authors provide a satisfactory explanation for, and discussion of, any heterogeneity observed in the results of the review?**	
For Yes:<br>X There was no significant heterogeneity in the results<br>☐ OR if heterogeneity was present the authors performed an investigation of sources of any heterogeneity in the results and discussed the impact of this on the results of the review	Yes<br>☐ No
**15. If they performed quantitative synthesis did the review authors carry out an adequate investigation of publication bias (small study bias) and discuss its likely impact on the results of the review?**	
For Yes:<br>performed graphical or statistical tests for publication bias and discussed the likelihood and magnitude of impact of publication bias	Yes<br>☐ No<br>☐ No meta-analysis conducted

16. Did the review authors report any potential sources of conflict of interest, including any funding they received for conducting the review?	
For Yes:<br>X The authors reported no competing interests OR<br>☐ The authors described their funding sources and how they managed potential conflicts of interest	**Yes**<br>☐ No

Table S2. Results of the articles selected in the systematic review.

Authors, year and country Design	Sample size	Criteria used to define remission	Probiotic used and length of the therapy	Control used and length of the therapy	Results
Fujimori et al.<br>2009<br>Japan<br>Randomized clinical trial	120	UC disease activity index ≤2.	The probiotic group ingested one daily capsule consisting of *Bifidobacterium longum* 2 × 10^9 colony-forming units and the prebiotic group ingested daily doses of 8.0 g of psyllium.<br>For 4 weeks.	The prebiotic group ingested daily doses of 8.0 g of psyllium.<br>The synbiotic group underwent both treatments.<br>For 4 weeks.	The total estimates of the inflammatory bowel disease questionnaires improve within the groups at the end of the trial (probiotics 162 to 169, NS; prebiotics 174 to 182, NS; synbiotics 168 to 176, p = **0.03**). The individual scores improve as follows: probiotics, emotional function (p = **0.03**); prebiotics, intestinal function (p = **0.04**); and synbiotics, systemic and social functions (p = **0.008** and p = **0.02**).
Sood et al.<br>2009<br>India<br>Randomized clinical trial	147	UC disease activity index ≤2.	*DSF* , a combination of probiotics.<br>Twice a day for 12 weeks.	Identical placebo appears for 12 weeks.<br>Twice a day for 12 weeks.	At week 12, there were 33 patients who received *DSF* (42.9%) and achieved remission, compared to 11 patients who received placebo (15.7%) (p < **0.001**).
Matthes el al.<br>2010<br>Germany	90	UC disease activity index ≤2.	A 40 ml, 20 ml, or 10 ml enema containing *Escherichia coli Nissle* 1917 (10^8 viable organisms/ml) od for at least 2 weeks.	Identical placebo twice a day for 12 weeks.	It was not significantly higher in the EcN group than in the placebo group (p = **0.4430**, 2-sided).

Randomized double-blind clinical trial					
Ng SC<br><br>2010<br>UK<br><br>Randomized double-blind clinical trial	28	UC disease activity index ≤2.	Two envelopes containing *DSF* (900 billion bacteria/sachet) bd for 8 weeks.	Identical placebo twice a day for 8 weeks.	In *DSF* treated patients, the expression of DC TLR-2 decreased ($p < \mathbf{0.05}$), the production of IL-10 increased, and the production of IL-12p40 decreased ($p < \mathbf{0.005}$); 10/14 patients on *DSF* showed a clinical response. Corticosteroids also resulted in increased IL-10 and reduced IL-12p40 production by DC. Conversely, in patients on placebo, the expression of TLR-2 and intensity of staining for IL-12p40 and IL-6 increased (all with $p < \mathbf{0.05}$);<br>5/14 patients on placebo showed a clinical response (p = NS).
Tursi et al.<br><br>2010<br>Italy<br><br>Randomized clinical trial	144	UC disease activity index ≤2.	For 8 weeks with *DSF* at a dose of 3.6 billion CFUs/day (71 patients).	For 8 weeks with placebo.	Remission was higher in the *DSF* group than in the placebo group (47.7% vs. 32.4%; $p = \mathbf{0.069}$, CI_{95} (%) 0.36-0.60; ITT $p = \mathbf{0.132}$, CI_{95} (%) 0.33-0.56). Eight patients with *DSF* (11.2%) and nine patients with placebo (12.3%) reported mild side effects.
Steed et al.<br><br>2010<br>UK<br><br>Randomized double blind trial	35	The clinical status was scored and rectal biopsies were obtained at baseline, and at 3 and 6 month intervals.	The patients received 2×10^{11} viable lyophilized *B. longum* in a gelatin capsule and a sachet containing 6 g of Synergy I (Orafti, Have, Belgium), twice a day for 6 months,	For 6 months with placebo.	There were significant improvements in clinical outcomes with the consumption of synbiotics, with reductions in both Crohn's Disease activity rates ($p = \mathbf{0.020}$) and histological scores ($p = \mathbf{0.018}$)
Benjamin et al.<br><br>2011<br>UK<br><br>Randomized	103	Crohn's Disease Acti vity Index (CDAI).	15 g/day fructo-oligosaccharides for 4 weeks.	Non-prebiotic placebo for 4 weeks.	There was no significant difference in the number of patients achieving a clinical response between the FOS and placebo groups in the ITT analysis (12 [22%] vs 19 [39%], $p = \mathbf{0.067}$).

double-blind clinical trial					
Ishikawa et al.<br><br>2011<br>Japan<br><br>Randomized clinical trial	41	The colonoscopic index and the amount of myeloperoxidase in a wash solution will be used as indexes of disease activity.	*Bifidobacterium breve Yakult* strain, a probiotic contained in bifidobacteria-fermented milk, and galacto-oligosaccharide (GOS) as synbiotic.<br><br>1 g of the probiotic powder (10^9 CFUs/g) three times a day, and 5.5 g of GOS once a day for one year.	The subjects in the control group were treated as usual on the basis of medical background (salazosulfapyridine, mesalazine, steroids).	The administration of the live strain of *B. breve Yakult* and GOS can improve the clinical condition of patients with UC.
Wildt et al.<br><br>2011<br>Denmark<br><br>Randomized double-blind clinical trial	32	Activity index of simple clinical colitis > 4 and/or endoscopic index ≥2.	Two capsules of Probio-Tec AB-25 (Chr. Hansen A / S, Hoersholm, Denmark) (1.25×10^{10} colony-forming units/capsule of *Lactobacillus acidophilus* LA - 5 and *Bifidobacterium animalis* BB - 12) tds for 52 weeks.	Three times a day of placebo with identical appearance for 52 weeks.	Five patients (25%) in the Probio-Tec AB-25 group and one patient (8%) in the placebo group maintained remission after 1 year of treatment (p = **0.37**). The median time to relapse was 125.5 days (range = 11–391 days) in the probiotic group, and 104 days (range = 28–369 days) in the placebo group, respectively, (p = **0.683**). Overall, Probio-Tec AB-25 was well tolerated.
Bourreille et al.<br><br>2013<br>France<br><br>Randomized double-blind clinical trial	165	Crohn's Disease activity index > 220, or 150-220 with an increase of ≥ 70 over baseline, or need for surgery or new medical therapy.	1 g *Saccharomyces boulardii*/ day for 52 weeks.	Identical appearing placebo for 52 weeks.	Crohn's Disease relapsed in 80 patients, 38 in the *S. boulardii* group (47.5%) and 42 in the placebo group (53.2%, no significant difference: p = **0.5**).
Petersen A. et al.<br><br>2014<br>Denmark<br><br>Randomized double-blind clinical trial	74	Rachmilewitz clinical activity index ≤4.	100 mg *Escherichia coli Nissle* 1917 ($2.5\text{-}25 \times 10^9$ viable organisms/capsule) for 4 days, then bd for 45 days.<br><br>The patients were assigned to Ciprofloxacin or placebo for 1 week, followed by EcN or placebo for 8 weeks. The 4 treatments were administered as complementary treatments.	Identical placebo for 8 weeks.	In the group that received placebo/EcN, fewer patients (54%) achieved remission compared to the group that received placebo/placebo: 89%, p **< 0.05**. Among the patients treated with Cipro/placebo and Cipro/EcN, 78% and 66% achieved remission, respectively. In addition, the placebo/EcN group had the highest number of withdrawals, 11 out of 25 (44%), compared with 15 out of 75 (20%) in any of

					the other groups, p **< 0.05.** The indication of lack of mucosal healing was found in the placebo/Nissle group, since only 4 (29%) of the 14 patients who completed the study did not report blood in the stool at week 12 (p **< 0.02),** compared to 63%, 67%, and 65% in the groups treated with Cipro/Nissle, Cipro/placebo and placebo/placebo, respectively.
Fedorak R. et al.<br><br>2015<br>Canada<br><br>Randomized double-blind clinical trial	98	Endoscopic: Rutgeerts score.	A package containing *DSF* (900 billion bacteria/sachet) bd for 3 months.<br>The groups that received 1 envelope of *DSF* (comprising 4 strains of *Lactobacillus*, 3 strains of *Bifidobacterium*, and 1 strain of *Streptococcus salivarius*, *thermophilus* subspecies).	Identical placebo twice a day for 3 months.	At day 90, the proportion of patients with severe endoscopic lesions did not differ significantly between *DSF* (9.3%) and placebo (15.7%, p **= 0.19**). The proportions of patients with non-serious injuries at day 90 who had severe endoscopic recurrence at day 365 were 10.0% in the early *DSF* group (they were given *DSF* during the full 365 days) and 26.7% in the *DSF* late group (they were given *DSF* from days 90 to 365) (p **= 0.09**).<br>The patients who received *DSF* had reduced levels of inflammatory cytokines in the mucosa compared to placebo at day 90 (p **< 0.05**). The activity index of Crohn's Disease and the quality of life scores of the inflammatory bowel disease were similar in the 2 groups.
Yoshimatsu et al.<br><br>2015<br>Japan<br><br><br>Randomized double-blind clinical trial	60	The clinical symptoms were evaluated monthly or on the exacerbation of symptoms or need for additional medication.	The patients were randomized to receive 9 Bio-Three tablets/day (Bio-Three group) or 9 placebo tablets/day (2 mg *Streptococcus faecalis* T - 110, 10 mg *Clostridium butyricum* TO - A, 10 mg *Bacillus mesentericus* TO - A) tds for 12 months.	Placebo for 12 months.	The relapse rates in the Bio-Three and placebo groups were, respectively, 0.0% vs. 17.4% at 3 months (p = 0.036), 8.7% vs. 26.1% at 6 months (p = 0.119), and 21.7% vs. 34.8% (p = 0.326) at 9 months. At 12 months, the remission rate was 69.5% in the Bio-Three group and 56.6% in the placebo group (p = **0.248**).
Tamaki H. et al.	56	UC disease activity	One sachet containing *Bifidobacterium longum* 536 (BB536) (2-3 × 10^{11} viable organisms/sachet) three times a day for 8 weeks.	Placebo for 8 weeks.	In total, 63% of the patients who received BB536 showed remission at week 8

2016<br>Japan<br><br>Randomized double-blind clinical trial		index ≤2.			compared to 52% of those who received placebo. We observed a significant decrease in the UCDAI scores in the BB536 group (p **< 0.01**), while there was no significant decrease in the placebo group (p **= 0.88**).
Matsuoka et al.<br><br>2018<br>Japan<br><br>Randomized double-blind clinical trial	195	The primary efficacy endpoint was relapse-free survival (relapse: rectal bleeding score ≥ 2 on the Sutherland disease activity index scale for 3 consecutive days and/or initiation of remission induction therapy due to worsening of UC).	One pack of BFM fermented milk per day [*Bifidobacterium breve Yakult* strain (10 billion bacteria) and *Lactobacillus acidophilus* (1 billion bacteria)].<br><br>For 48 weeks.	Placebo.<br><br>For 48 weeks.	Relapse-free survival was not significantly different between the BFM and placebo groups (p = **0.643**; Risk Ratio = 1.16; 95% CI = 0.63-2.14, log-rank test), nor was the incidence of relapse. Therefore, the study was discontinued for lack of efficacy.<br><br>Furthermore, the incidence of relapse was not significantly different (p = **0.651**) between the BFM (22.7%) and placebo (20.0%) groups.
Su H., Kang et al.<br><br>China<br>2018<br><br>Randomized clinical trial	123	Clinical efficacy: recovery, symptoms and clinical signs disappeared after treatment, routine stool examination was negative, microscopic ulcer healed, mucosal recovery was observed.	Probiotics: *Bifidobacterium Lactobacillus* triple tablets, at a dose of 4 x 500 mg per time, 2 times a day. Glucocorticoids: prednisone, at an initial dose of 0.75-1.0 mg/kg/day and gradually stopped in 3-4 months.	The patients in the control group were treated with routine treatment of oral sulfasalazine. At the same time, a total of 40 healthy individuals were selected to serve as the healthy group (received no treatment). 3-4 months.	After treatment, the number of intestinal flora in the treatment group reached that of the healthy individuals. The treatment efficiency of the treatment group was significantly higher than that of the control group, and the infection rate of the control group was significantly higher than that of the treatment group (p **< 0.05).**

Bjarnason et al.<br><br>2019<br>UK<br><br><br>Randomized double-blind clinical trial	143	The difference in change in the IBD Quality of Life (QoL) Questionnaire results between probiotic vs. placebo at week 4.<br>The secondary outcome measures included analyses of the change in laboratory findings, including Faecal Calprotectin (FCAL).	Probiotic (Symprove™, Symprove Ltd, Farnham, United Kingdom)<br><br>*Lactobacillus rhamnosus* NCIMB, 30174, *Lactobacillus plantarum* NCIMB 30173, *Lactobacillus acidophilus* NCIMB 30175, and *Enterococcus faecium* NCIMB 30176 in a water-based suspension of barley extract each with 50 ml/dose containing about 10 billion live bacteria.<br><br>4 weeks.	Placebo.<br><br>4 weeks.	There were no significant differences in the IBD-QOL scores between the placebo and the probiotic groups.<br><br>However, the differences in FCAL between patients with UC before and after probiotics versus placebo approached statistical significance with p = **0.076.**
Kamarlı et al.<br><br>2019<br>Turkey<br><br>Randomized clinical trial	40	The clinical activity was determined using the Truelove-Witts Clinical Activity Index, and the endoscopic activity was determined using the Ulcerative Colitis En doscopic Index of Severity (UCEIS).	The **synbiotic preparation** was composed of six probiotic strains ($3x10^9$ CFUs)-*Enterococcus faecium*, *Lactobacillus plantarum*, *Streptococcus thermophilus*, *Bifidobacterium lactis*, *Lactobacillus acidophilus*, *Bifidobacterium longum*, and fructo-oligosaccharide (225 mg/tablet)<br><br>For 8 weeks.	The placebo product had the same taste and appearance as the original product.<br><br>For 8 weeks.	The serum C-Reactive Protein (CRP) and sedimentation values in the synbiotic group were statistically significant (p = **0.003**). In both groups, a statistically significant improvement was observed in the clinical and endoscopic activity levels at the end of the treatment (synbiotic: p = 0.001 and p = **0.002**, respectively; control: p = **0.005** and p = **0.001**, respectively).
Sánchez-Morales et al.<br><br>2019<br>Mexico<br><br>Randomized clinical trial	34	The clinical activity was determined using the Truelove-Witts Clinical Activity Ind ex.	6 strains of probiotics (*Lactobacillus plantarum*, *Lactobacillus sacidophilus*, *Lactobacillus rhamnosus*, *Lactobacillus bifidus*, *Lactobacillus casei*, and *Bifidobacterium infantis*), at doses of 4×10^7 CFUs, before breakfast.<br><br>For 3 months.	Placebo: Nutritional treatment.<br><br>For 3 months.	An improvement was found in the disease activity (52.9% vs. 23.5%, p = **0.07**) and in the histologic index (82.3% vs. 41.1%, p = **0.03**) in the patients treated with probiotics compared to the control group.

Table S3. Concomitant Medication in Included Studies.

Trial	Concomitant medication
**Fujimori 2009**	*Excluded*: It does not indicate
	Permitted: aminosalicylates and prednisolone
**Sood 2009**	*Excluded*: Oral glucocorticosteroids within 4 weeks of inclusion. Antibiotics within 2 weeks of inclusion. Topical mesalazine or glucocorticosteroids within 7 days of inclusion. NSAIDs. Antidiarrhoeal agents.
	Permitted: Stable dose mesalazine and thiopurines.
**Matthes 2010**	*Excluded*: Topical glucocorticosteroids or aminosalicylates within 2 weeks of inclusion. Immunosuppressants within 90 days of inclusion. Antibiotics or sulphonamides during the study.
	Permitted: Oral aminosalicylates or glucocorticosteroids at stable dose for 2 weeks prior to inclusion.
**Ng 2010**	*Excluded*: Antibiotics within 2 weeks of inclusion. Alteration in dose of topical 5-ASA or steroids within 7 days of inclusion. Alternative probiotics.
	Permitted: Mesalazine (stable for 4 weeks prior to inclusion). Thiopurines (stable for12 weeks prior to inclusion).
**Tursi 2010**	*Excluded*: Oral glucocorticosteroids within 4 weeks of inclusion. Antibiotics within 2 weeks of inclusion. Topical 5-ASA or steroids within 1 week of inclusion. Alternative probiotics within 2 weeks of inclusion. NSAIDs within 1 week of inclusion.
	Permitted: 5-ASA (stable dose for 4 weeks prior to inclusion). Azathioprine or 6-mercaptopurine (stable for at least 3 months prior to inclusion).
**Steed 2010**	*Excluded*: It does not indicate
	Permitted: Patients were also requested to continue on stable doses of conventional CD medication
**Benjamin 2011**	*Excluded:* anti-tumour necrosis factor agents in the preceding 12 weeks; antibiotics, probiotics or prebiotics in the preceding 4 weeks; rectal preparations during the preceding 2 weeks; and any non-steroidal anti-inflammatory drugs during the preceding week. hange in dose of immunosuppressant within 12 weeks and oral 5-aminosalicylic acid or steroids within 4 weeks. The maximum permissible steroid dose was 20 mg/day
	Permitted: Standard medical care based on physicians' discretion
**Ishikawa 2011**	*Excluded:* It does not indicate
	Permitted: Salazosulfapyridine, mesalazine, steroids
**Wildt 2011**	*Excluded*: Treatment with all UC medications bar stable dose 5-aminosalicylates.
	Permitted: 5-ASA at stable dose for at least 4 weeks prior to inclusion.
**Bourreille 2013**	*Excluded*: Immunosuppressive treatments or anti TNFα within 3 months of inclusion. Probiotics, antibiotics, or antifungal treatments for more than 2 weeks.
	Permitted: Glucocorticosteroids or budesonide and/or aminosalicylates according to the preference of each investigator to achieve remission, then weaned off within 12 weeks of inclusion.
**Petersen 2014**	*Excluded*: Systemic glucocorticosteroids or biologic therapy.
	Permitted: Standard medical care based on physicians' discretion. Topical glucocorticosteroids.
**Fedorak 2015**	*Excluded*: Anti-TNF within 8 weeks of resection.
	Permitted: Codeine, loperamide, diphenoxylate, and cholestyramine.
**Yoshimatsu 2015**	*Excluded*: Granulocyte-monocyte adsorptive apheresis, thiopurines, cyclosporine, antibiotics.
	Permitted: Stable dose mesalazine, salazosulfapyridine or steroids for 4 weeks prior to inclusion.
**Tamaki 2016**	*Excluded*: Antibiotics within 2 weeks of inclusion. Topical 5-ASA or glucocorticosteroids within 7 days of inclusion. NSAIDs and antidiarrhoeal drugs during the study period.

	Permitted: 5-ASA, prednisolone and thiopurines at stable dose for 4 weeks prior to inclusion.
	Permitted: Pre-treatment with sulfasalazine and glucocorticosteroid
	Permitted: No concomitant medication for UC was allowed.
**Matsuoka 2018**	*Excluded: 5-ASA treatment, glucocorticoids, immunomodulators/immunosuppressants, cytapheresis, and antibiotics and antibacterial agents.*
	Permitted: Restricted treatments were allowed with conditions and included standard treatments for UC if patients were taking them at the time of enrollment
**SU 2018**	*Excluded: patients who were allergic to probiotics and glucocorticoids*
	Permitted: glucocorticoids
**Bjarnason 2019**	*Excluded*: steroids (prednisolone > 4 mg/day) and biologics
	Permitted: treatment with a 5-aminosalicylic preparation or low dose Azathioprine (1 mg/kg)
**Kamarlı 2019**	*Excluded*: administered corticosteroids or biological therapy 4 weeks before the study, who were found to have a concurrent enteric infection, who used probiotic and/or synbiotic preparations and antibiotics 2 weeks before the study, pregnant and breastfeeding women, patients with end-stage liver and renal failure, and those with sensitivity to probiotics and/or synbiotics.
	Permitted: mesalazine, azatioprina
**Sánchez-Morales 2019**	*Excluded*: TNF-alpha antagonists
	Permitted: mesalazine (2 g per day on average); none of them wasreceiving glucocorticoids or other immunosuppressant at time to enter the study.

Table S4. Outcomes of randomized controlled trials evaluating the effects of probiotics on IBDs.

**Study**	**Subject**	**p value**
Fujimori et al. (2009)	UC	0.03
Sood et al. (2009)	Active UC	0.01
Steed et al. (2010)	Active CD	0.01
Matthes et al. (2010)	Active UC	0.04
Ng SC (2010)	Active UC	0.05
Tursi et al. (2010)	UC under ASA treat	0.06
Benjamin (2011)	Active CD	0.06
Ishikawa et al. (2011)	Mild to moderate UC	0.05
Wildt et al. (2011)	Left-side Inactive UC	0.3
Bourreille et al. (2013)	CD treat with steroids	0.37
Petersen et al. (2014)	Active UC	0.05
Yoshimatsu et al. (2015)	Inactive UC	0.2
Fedorak et al. (2015)	CD after surgery	0.8
Tamaki et al. (2016)	Mild to moderate UC	0.03

Matsuoka (2018)	Inactive UC	0,6
Su H (2018)	Active CD	0.05
Bjarnason (2019)	Active CD	0.5
	Active UC	0.5/0.076
Kamarlı (2019)	Active UC	0.001
Sánchez-Morales (2019)	Active UC	0.004

Figure S1. Single species versus mixture for the remission of UC.

Study or Subgroup	Probiotics Events	Probiotics Total	Control Events	Control Total	Weight	Odds Ratio M-H, Fixed, 95% CI	Risk of Bias A	B	C	D	E	F	G
**5.1.1 Single Species**													
Fujimori 2009	29	40	54	80	9.5%	1.27 [0.55, 2.93]	+	?	+	?	?	+	?
Matthes 2010	41	70	13	20	8.0%	0.76 [0.27, 2.14]	+	+	+	+	+	+	+
Petersen 2014	15	25	46	75	8.8%	0.95 [0.37, 2.39]	+	+	+	+	+	+	−
Tamaki 2016	10	28	13	28	8.0%	0.64 [0.22, 1.87]	+	?	+	+	−	+	+
**Subtotal (95% CI)**		**163**		**203**	**34.4%**	**0.92 [0.57, 1.48]**							
Total events	95		126										
Heterogeneity: Chi² = 1.14, df = 3 (P = 0.77); I² = 0%													
Test for overall effect: Z = 0.34 (P = 0.73)													
**5.1.2 Mixture Species**													
Kamarlı 2019	10	20	14	20	6.7%	0.43 [0.12, 1.57]	+	?	?	?	+	+	+
Ng SC 2010	4	14	9	14	6.2%	0.22 [0.05, 1.09]	?	?	+	+	+	+	−
Sanchez 2019	8	17	13	17	6.6%	0.27 [0.06, 1.19]	+	−	+	?	+	+	?
Sood 2009	44	77	59	70	25.4%	0.25 [0.11, 0.55]	+	+	+	+	−	+	?
Tursi 2010	40	71	50	73	20.7%	0.59 [0.30, 1.17]	+	?	+	+	+	+	?
**Subtotal (95% CI)**		**199**		**194**	**65.6%**	**0.38 [0.24, 0.58]**							
Total events	106		145										
Heterogeneity: Chi² = 3.43, df = 4 (P = 0.49); I² = 0%													
Test for overall effect: Z = 4.44 (P < 0.00001)													
**Total (95% CI)**		**362**		**397**	**100.0%**	**0.56 [0.41, 0.77]**							
Total events	201		271										
Heterogeneity: Chi² = 11.79, df = 8 (P = 0.16); I² = 32%													
Test for overall effect: Z = 3.59 (P = 0.0003)													
Test for subgroup differences: Chi² = 7.54, df = 1 (P = 0.006), I² = 86.7%													

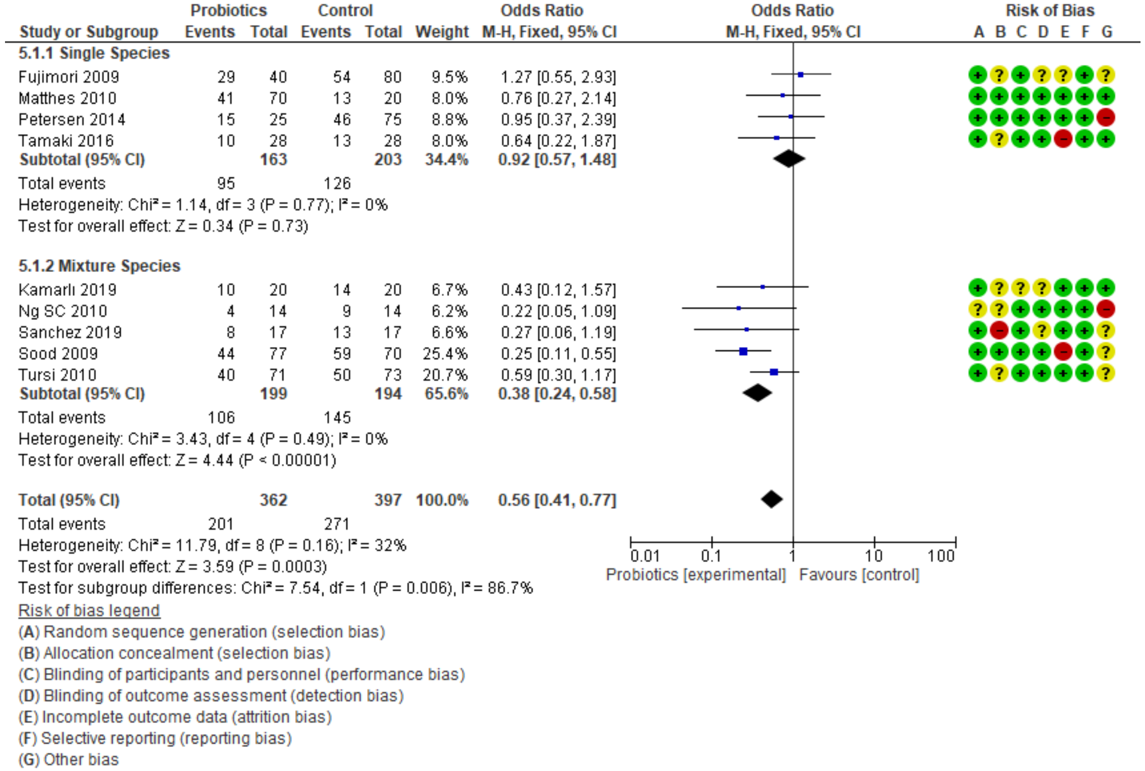

Risk of bias legend
(A) Random sequence generation (selection bias)
(B) Allocation concealment (selection bias)
(C) Blinding of participants and personnel (performance bias)
(D) Blinding of outcome assessment (detection bias)
(E) Incomplete outcome data (attrition bias)
(F) Selective reporting (reporting bias)
(G) Other bias

Figure S2. Single species versus mixture for the remission of CD.

Study or Subgroup	Probiotics Events	Probiotics Total	Control Events	Control Total	Weight	Odds Ratio M-H, Fixed, 95% CI	A	B	C	D	E	F	G
**6.1.1 Single Species**													
Benjamin 2011	12	54	19	49	23.9%	0.45 [0.19, 1.07]	+	?	+	+	+	+	+
Bourreille 2013	46	84	39	81	27.7%	1.30 [0.71, 2.40]	+	−	+	+	?	+	+
Steed 2010	8	13	5	11	3.2%	1.92 [0.38, 9.80]	+	?	+	+	−	?	−
Su 2018	28	43	32	40	17.8%	0.47 [0.17, 1.26]	+	−	?	−	+	+	?
**Subtotal (95% CI)**		**194**		**181**	**72.7%**	**0.84 [0.55, 1.29]**							
Total events	94		95										
Heterogeneity: Chi² = 6.30, df = 3 (P = 0.10); I² = 52%													
Test for overall effect: Z = 0.78 (P = 0.43)													
**6.1.2 Mixture**													
Fedorak 2015	32	59	39	60	27.3%	0.64 [0.31, 1.33]	+	?	+	+	−	+	?
**Subtotal (95% CI)**		**59**		**60**	**27.3%**	**0.64 [0.31, 1.33]**							
Total events	32		39										
Heterogeneity: Not applicable													
Test for overall effect: Z = 1.19 (P = 0.23)													
**Total (95% CI)**		**253**		**241**	**100.0%**	**0.79 [0.55, 1.14]**							
Total events	126		134										
Heterogeneity: Chi² = 6.73, df = 4 (P = 0.15); I² = 41%													
Test for overall effect: Z = 1.27 (P = 0.20)													
Test for subgroup differences: Chi² = 0.42, df = 1 (P = 0.52), I² = 0%													

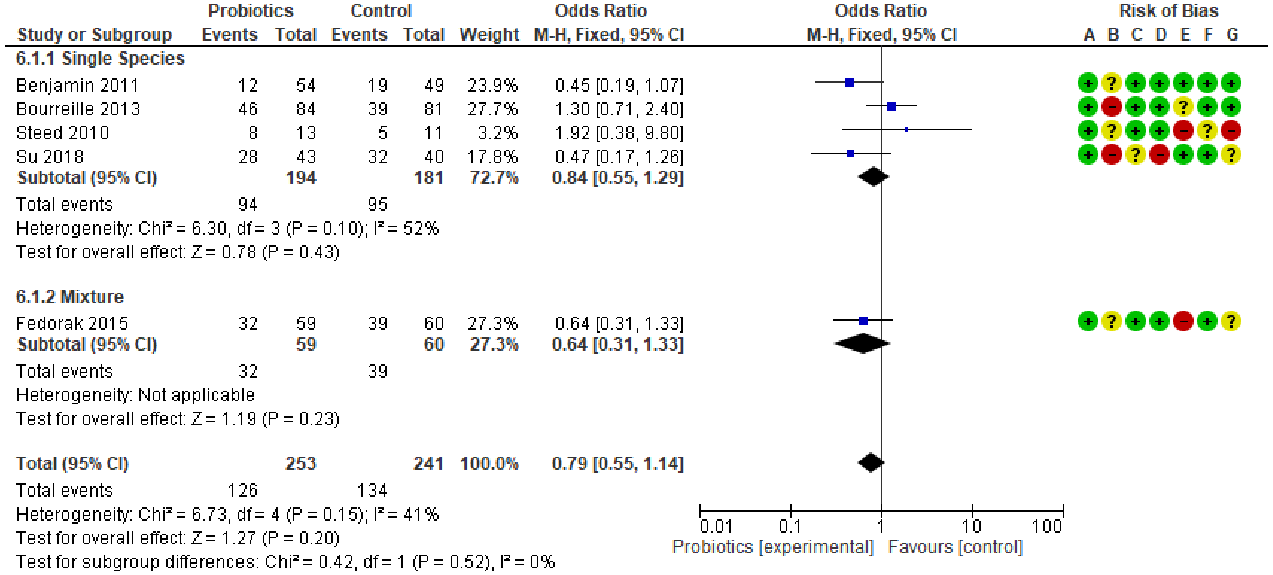

Risk of bias legend
(A) Random sequence generation (selection bias)
(B) Allocation concealment (selection bias)
(C) Blinding of participants and personnel (performance bias)
(D) Blinding of outcome assessment (detection bias)
(E) Incomplete outcome data (attrition bias)
(F) Selective reporting (reporting bias)
(G) Other bias
